# Apologetic

**Published:** 1868-11-01

**Authors:** 


### *EDITORIAL.*

### *Apologetic.*

The Editor begs pardon of the readers of the Journal for having recently referred to a semi (or more) quack institution and its "organ" in the city of Cincinnati. At the time the paragraphs referred to were written, he supposed each to have a certain amount of respectability. Recent developments have shown that neither is possessed of this desirable quality.

This reminds us of an anecdote we heard many years ago from the lips of the Rev. Lyman Beecher, the father of all the Beechers. It was in the quaint, old-fashioned town of Middlebury, Vermont, before a college literary society with which we were then connected. The old gentleman, in paternal style, was advising us to weigh well the character of an antagonist before we engaged in a controversy with him. "Once upon a time," said he, "I was walking along the road in the evening with an old black letter volume under my arm, the contents of which I had, just before, been carefully conning. A few steps in front of me I saw a little black and white animal crossing the way. Without a moment's reflection I hurled the whole quarto volume at him, and —*got teh worst of the bargain!*"

The editor regrets exceedingly that a few weeks since he did not happen to recollect the moral conveyed in Dr. Beecher's entertaining experience. We promise not to defile our pages with any more of the odor of the *Cincinnati Medical Repertory*.

